# Alcoholism,—A Crime or a Disease1Read at the thirty-third annual meeting of the Medical Association of Central New York, held at Rochester, N. Y., October 16, 1900.

**Published:** 1901-02

**Authors:** F. E. Fronczak

**Affiliations:** Buffalo, N. Y.


### ALCOHOLISM,—A CRIME OR A DISEASE.[1]

By F. E. FRONCZAK, A. M., M. D., Buffalo, N. Y.

FOR some time past, voices have been raised and heard in various parts of this country, and also in Europe, on the important and ever interest-attracting question of alcoholism. Is an alcoholic a criminal, or a sick man? Is a chronic drunkard always hovering on the border of delirium tremens, subject to the penal code of our commonwealths, or is he a candidate for a cot in a hospital ward? Is a man, who under the influence of liquor commits any criminal act liable to punishment, or should he, as the person suffering from mental aberration, whether temporary or of longer standing, be committed to an asylum for inebriates for treatment? These are questions which stagger many a medico-legal specialist.

---

1. Read at the thirty-third annual meeting of the Medical Association of Central New York, held at Rochester, N. Y., October 16, 1900.

The ninth congress of Polish physicians and scientists, at its last meeting in July, at Cracow, declared the question to be one deserving the highest consideration, and at its next congress at Lemberg in 1903, will devote most of its time to the proper steps tending to the solution of this matter.

Alcoholism, and its victims, the drunkards, cost every community large sums annually. Laws of all kinds have been passed bearing on the liquor traffic and all kinds of means have been taken for its suppression and limitation of its consequences. Families have been ruined, and crimes, aye murders, have been committed in drunken frenzy. If smallpox and cholera are quarantined, if we try to discover the causes which produce tuberculosis or typhoid fever, why should we not try to prevent causes which perpetuate alcoholism. If any of the above mentioned diseases attack a person, the patient undergoes treatment, why should not an alcoholic also be treated?

This is the age of prophylaxis. The highest and most eminent branch of the medical science is the teaching of hygiene, or preservation of health and prevention of disease. The preventive measures for the fracture of a limb, I suppose the surgeon would name: "Do not fracture it." The preventive measures for alcoholism, I answer, "Do not expose yourself to alcoholic influences." And this we must leave to the solons of the legislatures of our different states and the nation, who are supposed to pass such laws as will bring about this condition. But when one does expose himself to the morbid conditions following alcoholic indulgence, whether this condition is chronic or temporary, I believe the members of our profession owe it a duty to themselves and to mankind to step in and use their power to bring the patient to his former condition, as much as this is possible.

Alcoholism is classed among the nervous and mental diseases, and the pathology, as studied by various eminent authors' shows most marked changes in the nervous system; various nerve cells are changed and disintegrated, capillaries are dilated in the entire central nervous apparatus, in one word, there is an abnormal condition in that system after each and every alcoholic debauchery. These are the physical conditions we are to rectify, and not simply the moral one which the penal code provides for the treatment of this so-called crime.

Gentlemen, in my annual report for 1900, as physician of the Erie County Penitentiary, I said:

Drunkenness is not a crime, it is a disease, and should be treated as such. This is an opinion of men of eminence in law and medicine, and therefore let us begin to treat it as such, in this the closing year of the nineteenth century of our era. Insanity was considered a crime but a short time ago, but now the civilised world considers it a morbid condition of the mind, and provides proper hospitals for its cure. Let us, therefore, give the alcoholics, like the insane, proper surroundings and proper treatment and cure. We do not liberate the insane before he is considered cured by proper authorities; let us therefore, on the same basis, put alcoholics where they properly belong. I recommend this to your kind consideration.

What I said in that report, Mr. Chairman, I repeat. Give the alcoholics, meaning the chronic drunkards, the men who compose the majority of the prisoners on the blotters at the police stations, and who occupy the greater majority of the cells in the penal institutions, proper hospitals, proper surroundings, and proper treatment and care, and a large percentage of chronic alcoholism will disappear. Alcoholism, like smallpox through vaccination, or typhoid fever through hygienic prophylactic measures, will slowly cease to be an endemic disease in certain localities, and many a medico-legal case will thereby be eliminated from the dockets of our criminal courts.

508 Fillmore Avenue.

